# How to Implement Automotive Fault Diagnosis Using Artificial Intelligence Scheme

**DOI:** 10.3390/mi13091380

**Published:** 2022-08-24

**Authors:** Cihun-Siyong Alex Gong, Chih-Hui Simon Su, Yu-Hua Chen, De-Yu Guu

**Affiliations:** 1 Department of Electrical Engineering, School of Electrical and Computer Engineering, College of Engineering, Chang Gung University, Taoyuan 33302, Taiwan; 2 Portable Energy System Group, Green Technology Research Center, College of Engineering, Chang Gung University, Taoyuan 33302, Taiwan; 3 Department of Neurosurgery, Chang Gung Memorial Hospital, Linkou, Taoyuan 33302, Taiwan

**Keywords:** artificial intelligence (AI), machine learning (ML), supervised learning, unsupervised learning, reinforcement learning, modeling, vehicle fault detection and diagnosis (VFDD), micromachined, sensor

## Abstract

The necessity of vehicle fault detection and diagnosis (VFDD) is one of the main goals and demands of the Internet of Vehicles (IoV) in autonomous applications. This paper integrates various machine learning algorithms, which are applied to the failure prediction and warning of various types of vehicles, such as the vehicle transmission system, abnormal engine operation, and tire condition prediction. This paper first discusses the three main AI algorithms, such as supervised learning, unsupervised learning, and reinforcement learning, and compares the advantages and disadvantages of each algorithm in the application of system prediction. In the second part, we summarize which artificial intelligence algorithm architectures are suitable for each system failure condition. According to the fault status of different vehicles, it is necessary to carry out the evaluation of the digital filtering process. At the same time, it is necessary to preconstruct its model analysis and adjust the parameter attributes, types, and number of samples of various vehicle prediction models according to the analysis results, followed by optimization to obtain various vehicle models. Finally, through a cross-comparison and sorting, the artificial intelligence failure prediction models can be obtained, which can correspond to the failure status of a certain car model and a certain system, thereby realizing a most appropriate AI model for a specific application.

## 1. Introduction

In recent years, due to the advancement of semiconductor technology, it has solved many existing hardware computing bottleneck problems, coupled with the advancement of 5G networks and the significant improvement of communication efficiency, resulting in the acceleration of artificial intelligence (AI) applications [1]. Internet of Things (IoT) applications are ubiquitous, and the realization of the Internet of Vehicles (IoV) in countries around the world has accelerated the trend of commercial operation [2]. Communication technologies, such as Industry 4.0/5.0 or 5G/6G, are also necessary or indispensable infrastructures for enterprises and industries to go digital. The key architecture of the IoT is the integration of a large number of smart sensors, signal acquisition devices, and edge computing processors [3,4]. The system under evaluation makes it possible to convert sensory signals into computable base data, which are then statistically or digitally filtered according to appropriate algorithms. The obtained characteristic data can be further identified and interpreted, and the characteristic data can also be transformed into the physical form to explain the surrounding environment of the system, and, finally, to realize the corresponding feedback control mechanism. With the advent of the era of the IoV and the rapid advancement of miniaturized process technologies such as contemporary MEMS, the importance of sensors in vehicle failure prediction has become indescribable, and this has led to a new wave of industrial revolutions such as the production of the IoV.

In addition, the system parts of the vehicle are composed of many rotating structures. Under the wear and tear of long-term driving and operation, the relevant components are prone to experiencing functional abnormalities due to reasons such as temperature, pressure, metal fatigue, and wear and tear [5]. However, today’s transportation-related command, control, communication, and intelligence systems are developing rapidly, and we still face numerous unpredictable risks [6]. In terms of past technology developments, fault diagnosis mainly relies on sensors to collect relevant physical analog signals [7]; then, from a large amount of data, according to field operations or the past, a reference data threshold (also known as the threshold value) or a specific data threshold is set to observe, compare, and determine subsequent adjustments. The trend value reference enables the system to perform diagnostic identification and analysis on the basis of calculation identification. If the vehicle health big data can be used to analyze and collect the dynamic characteristics of tires, chassis, and engines, the prediction system can continue to give feedback and early warnings, and compare the identification results with the characteristic data of normal conditions, so as to further realize the early diagnosis and prediction of faults, thereby greatly improving the driving safety of the vehicle [8]. In recent years, the commercial application of the IoV has attracted much attention, and vehicle failure prediction has become a key research topic. Therefore, if the abnormal diagnosis and prediction system can be implemented in the early stage of vehicle failure, and the types and symptoms of the failure can be predicted, traffic accidents caused by mechanical failures can be greatly reduced, and the threat to the lives of passers-by and property losses can be effectively reduced.

To achieve early failure prediction, the first approach is to build physics-based models, which are characterized by a physical description of how the machine works. Today, even though data-based approaches are primarily implemented, it may be more appropriate to opt for a physics-based model. Mathematically, this approach correlates vehicle component wear phenomena to the component’s useful life. Among the variables considered in the formulation of the physical–mathematical model, various physical quantities describe the thermal, mechanical, chemical, and electrical properties of the component being analyzed. Being able to describe their impact on machine health is a rather tricky task, as this type of solution requires a high level of domain knowledge. Once the model is established, there must be accurate sensors available in order to obtain the physical quantities considered and the assumed values being correlated as inputs during the analysis and modeling phases. The main advantage of this approach is that it allows the precise description of the output it provides since it is based on a physical description of the process. As for accuracy, it is closely related to the quality of data analysis and modeling by domain experts. On the other hand, its disadvantages are the complexity, high implementation cost, and high specificity of the system, which make it unlikely to be widely used and extended for commercial applications [9].

In addition, domain experts are starting to create knowledge-based models, as this approach aims to simulate the skills and behaviors of experts. Therefore, once the knowledge is embodied, it can be automatically copied and applied. Expert systems are programs that utilize expert knowledge in a specific domain and apply reasoning mechanisms to model ideas and provide support and practical solutions. The most common ways to implement such models are rule-based systems and fuzzy logic. Rule-based systems have the advantage of being easy to implement and interpretable, but their performance can be poor, especially when complex conditions need to be expressed or the number of rules is very large. Likewise, fuzzy logic allows the state of the system to be described by imitating the human decision-making process, making the formal process and description of the model more direct and intuitive. Even for expert systems, the final results are highly dependent on the level of quality and accuracy achieved by the model compared to physical models, and the results are highly specific [10].

Due to the rapid development of automotive active protection functions, traditional rule-based diagnostic systems have become very limited. Therefore, it is necessary to study more complex data-driven methods and use highly integrated (such as the use of sophisticated MEMS technology) sensors to match one another to find more effective solutions. At present, the application method of fault prediction is data-based models, which are also the main discussion in this paper. Data-based models can be mainly divided into the following categories: statistical approaches [11], stochastic approaches [12], and machine learning techniques [13,14,15]. Statistical and stochastic methods make it possible to deal with complex systems, but the accuracy of predictions varies over time. Applying statistical methods to predict, estimate, and optimize the average life of a system may be beneficial in certain specific situations related to the operation of mechanical components, such as the battery of an electric vehicle or the gear train of an automobile.

Machine learning, an important method used in artificial intelligence (AI), deals with creating algorithms that learn and improve performance based on the big data used. There are three types of machine learning algorithms currently in use: supervised learning, unsupervised learning, and reinforcement learning. The difference between these three algorithms depends on how each algorithm learns the dataset to determine predictions.

## 2. Overview of Machine and Deep Learning Methods

As mentioned in the Introduction Section, machine learning is an important approach to artificial intelligence (AI), which deals with creating systems that learn and improve performance based on the data they use. In unsupervised learning, the data are not labeled and the model is formulated so that it recognizes patterns and structures in the data by itself. In supervised learning, ML models use labeled training data. Supervised learning is the most commonly used machine learning in practical applications. Finally, reinforcement learning enables the system to learn through rules, trial, and error to discover the current situation that is closest to its actual environment usage. With regard to applications in the automotive industry, reinforcement learning has been the basis for the development of self-driving cars, which learn to recognize the surrounding environment (using data collected with GPS, sensors, etc.), and based on the driving conditions they have to actually face. Machine learning algorithms require an effective analysis of large amounts of historical data and real-time data inferred from multiple data streams (sensors and data collection systems) [16]. Therefore, the data-preprocessing stage has a significant impact on the performance of machine learning algorithms [17,18,19]. This section explores traditional machine learning methods and more advanced deep learning methods, both of which are commonly used for predictive maintenance in the automotive domain [20].

### 2.1. Unsupervised Learning

The first part introduces unsupervised learning. In many pen-type data, the machine learning method of manual data labeling (label) is not included. This method is used when there is not too much manpower to interpret the data, or there is no basic theory to interpret the data. It is one of the most effective application methods. There are roughly two forms of unsupervised learning, namely, clustering [21] and generation [22]. Clustering is the ability to separate data based on distance or similarity. For example, the K-means algorithm introduced in the subtitle of the next paragraph is a generative unsupervised learning algorithm, while generation is able to generate pictures or data we want through random data. The most classic algorithm is the GAN algorithm. Additionally, PCA is a subitem under K-means, so it is also a kind of grouping. Because the principal component analysis (PCA) is an application of data dimensionality reduction and has a wide range of applicability, it had to be included in this article.

#### 2.1.1. K-Means

The K-means algorithm originated from a vector quantization method in signal processing, and, now, it is often popular in the field of data mining as a clustering method [23]. The K-means algorithm is a classic technique in the clustering literature. It uses prototype-based clustering as the prototype of hidden layers in the RBF (radial basis function) method. Broadly speaking, the K-means algorithm proceeds as follows: At initialization, the m-cluster prototypes are set to m random training points. Then, each of the n data points is assigned the smallest Euclidean distance to the prototype it is in. The distribution points for each prototype are averaged in order to create a new cluster center. In other words, the centroids of the created clusters (clusters) are used to replace their old prototypes with new ones, repeating this process repeatedly to reach convergence. Convergence is achieved when the cluster assignment is unchanged from one iteration to the next [24].

#### 2.1.2. PCA (Principal Component Analysis, PCA)

The principal component analysis (PCA) is one of the oldest statistical analysis techniques. The method is suitable for the multidimensional analysis of data [25,26]. In general, it is not sufficient to study each factor individually, as it does not allow for the detection of possible dependencies between factors. To reduce the number of factors (components), the PCA constructs an input space (also called a representation space), whose dimension is, thus, smaller than the number of factors, where observations (points) are distributed as closely as possible to their distribution in the representation space. The similarity criterion is the total inertia of the scatterplot. Therefore, the PCA is a linear mapping method that maximizes the scatter plot [27]. The PCA dimensionality reduction algorithm, proposed by British mathematician Carl Pearson (1857–1936) in 1901 [28], uses data standardization to perform eigendecomposition on the covariance matrix [29], and obtains eigenvectors, which are the principal component part of the data [30]. The PCA is mainly used in linear regression [31], because there is usually an important linear relationship between the input and output of a function. Using a linear transformation method, the original data are divided into corresponding principal components, and less relevant information is removed.

#### 2.1.3. GANs (Generative Adversarial Networks)

Compared with other deep learning neural network methods, the GAN was developed only recently, and further research and applications began after the publishing of the paper by Ian Goodfellow in 2014 [32]. Generative adversarial networks consist of two parts—the generative network and discriminative network. These two learning models are outlined below.

The first is a generative model, which generates a real library similar to synthetic examples, with the goal of creating synthetic objects that cannot distinguish whether a particular object belongs to the original dataset or is even synthetically generated. For example, if we were to have a library of car images, the generative network would use the generative model to create synthetic examples of car images. As a result, we could have real and generated car image examples. The second network is the discriminative network model, since it has been trained on a dataset labeled with images, whether synthetic or fake. A discriminative model that takes inputs from any real paradigm generates a network that creates underlying data or synthetic objects, and tries to identify whether an item is real or fake. In a sense, the generative network can be regarded as a “counterfeiter” trying to create counterfeit banknotes, and the discriminant network can be regarded as a “policeman” trying to catch counterfeit banknotes. Therefore, the two networks are adversaries, and the training makes both adversaries better until an equilibrium is reached between them (equilibrium). When the discriminative network is able to correctly label the synthetic object as false, the generative network uses this fact to modify its weights in order to distinguish the network from which it would be difficult to classify the sample material generated from it. After the modification, the weights of the generator network are repeatedly updated to generate new samples from it. Over time, generative networks have become better at producing counterfeit products. Ultimately, the discriminator is unable to distinguish between real and synthetically generated objects. The generated objects can often be used to create large amounts of synthetic data and can play a role in data augmentation [33].

### 2.2. Supervised Learning

Supervised learning means algorithms are trained on input data that have been labeled as a specific output target. During its training phase, the system receives labeled datasets that represent the associations between the trained model and each specific input and output. The trained model is then distinguished from the training set and the test dataset. The purpose of the test dataset is to measure how well the algorithm performs on unlabeled data. The model is trained until it can identify potential relationships between new input data and output labels, allowing it to produce accurate predictions when presented with previously unknown data. The process of supervised learning and its common methods are described in more detail below.

#### 2.2.1. Linear Regression

Linear regression, also known as least square regression, is a statistical method used to predict the value of a continuous dependent variable based on the value of the independent variable. The dependent variable and the independent variable are also called the response variable and the explanatory variable, respectively. As the mathematical relationship between the response variable and the explanatory variable, linear regression assumes that there is a linear correlation between the response variable and the explanatory variable, which is represented by the line of best fit, also known as the regression line. The development of a linear regression model begins by expressing a linear relationship. Once the mathematical form is established, the next step is to estimate the parameters of the model through model fitting. This determines the line of best fit achieved through the least squares estimation with the aim of reducing the sum of square error (SSE). The final stage is to evaluate the model using the R-square or mean square error (MSE). The MSE is a measure that determines how close the line of best fit is to the actual value of the response variable. As a straight line, the regression line cannot pass through every point; it is an approximation of the actual value of the response variable based on the estimated value. The distance between the actual value of the response variable and the estimated value is the estimation error. In order to estimate the response variable as best as possible, the error between all points (represented by the sum of squared errors) must be minimized. The line of best fit is the line that results in the smallest sum of square errors. In other words, the MSE identifies the change between the actual and estimated values of the response variable provided by the regression line. The coefficient of determination (called R-squared) is the percentage of variation in the response variable that is predicted or explained by the explanatory variable, and has a value between 0 and 1. A value of R-squared equal to 0 indicates that the response variable cannot be predicted from the explanatory variable, while a value of R-squared equal to 1 can predict the response variable without any error. A value between 0 and 1 provides the percentage of successful predictions. In regression, more than two explanatory variables can be used simultaneously to predict the response variable, in which case it is called multiple linear regression [34].

#### 2.2.2. Decision Tree

In decision analysis methods, decision trees can be used to visually and explicitly represent decisions and decision making. Although a common tool in data mining for deriving strategies to achieve specific goals, it is also widely used in machine learning classification. It is a tree-structured classifier, where internal nodes represent the features of the dataset, branches represent decision rules, and each leaf node represents the outcome. In a decision tree, there are two nodes, a decision node and a leaf node. The decision nodes are used to make any decision and have multiple branches. The leaf nodes are the output vectors of these decisions and do not contain any further branches. To build the decision tree, the classification and regression tree algorithm (CART) is used. The decision tree only asks one question at a time, and then presents a “yes or no” according to the answer. It further splits the original decision tree into subtrees. These features are helpful for building complex split surfaces because they allow decision trees to relearn faster [24], but they are rarely used.

#### 2.2.3. SVM (Support Vector Machine)

**Figure 1:**
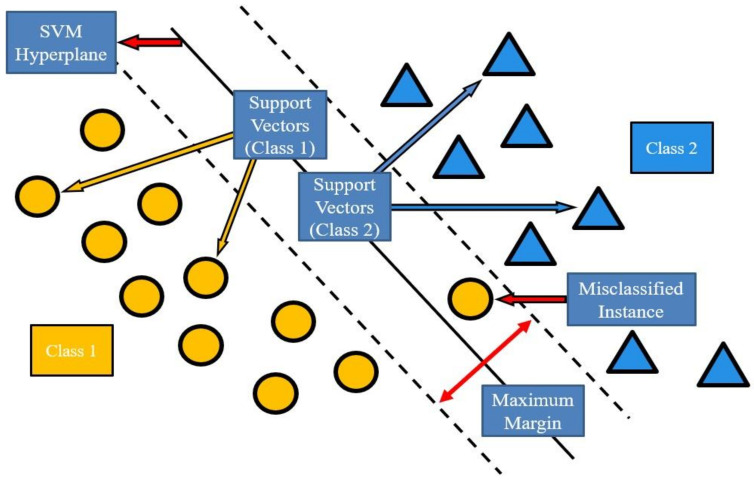
Schematic diagram of SVM.

The support vector machine (SVM) is a machine learning method for data classification, regression, and pattern recognition using the statistical learning theory. The main architecture of the SVM is composed of the Kernel function and hyperplane, where these two are also important factors that determine the effect and stability of SVM training. The aim is to find a decision boundary to maximize the margins between the two categories so that they are perfectly separated. The main function of the hyperplane is to map the feature vector of the data to a higher-dimensional space, and divide the data into two in this space, effectively distinguishing the category to which they belong. Another main theory of the SVM is the Kernel function, which can be classified by the hyperplane when the data are linearly separable. However, when the data cannot be classified linearly, it is necessary to convert and map the data to high dimensions through the Kernel function. The main purpose of this theory is to convert nonlinear to linear data and then to segment them. Figure 1 is a schematic diagram of the SVM. The support vector machine, which is commonly used in machine learning algorithms, has been proposed by researchers [35]. It has shown very good performance in solving small-scale data samples, nonlinear, high-dimensional models, and recognition ability. Therefore, it can effectively solve high-dimensional problems that often occur in the fault diagnosis of machine systems. In a recent study [36], a support vector machine combined with a deep learning architecture was proven to possess a good learning ability under many circumstances.

#### 2.2.4. Neural Network

In neural network algorithms, the supervised learning process is improved by continuously measuring the amount of errors in the output of a model and fine-tuning the system weights to approach closer to its predicted target accuracy. Neural-like networks are used in the field of machine learning and data science to imitate the neural structure and function of animal brains with mathematical models and algorithms to predict or estimate a mathematical function model. An artificial neural network, with a set of adjustable function weights, can be viewed as the correlation between neurons. In the process of a neural network calculation, if the single-layer neural network uses a larger number of neurons for the calculation, the recognition rate results are usually better. Generally speaking, when the number of hidden layers of the network exceeds 13 layers, it can be defined as a deep learning network. In fact, too many neurons can lead to a high power consumption and long recognition time. Regarding the reason why the learning effect of a multilayer neural network is better than that of single layer, Max Tegmark and Henry Lin put forward a hypothesis [37]: setting the hidden layer of deep learning as a symmetric structure is closer to the pattern in nature. Additionally, the number of neurons used can be reduced to achieve better performance. A neural network can be further subdivided into a feedforward neural network (FNN), recurrent neural network (RNN), and reinforcement neural network, according to the structure.

#### 2.2.5. LSTM (Long Short-Term Memory)

**Figure 2:**
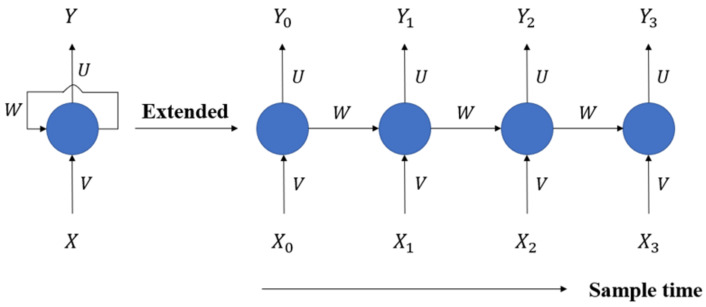
RNN neuron (**left**) unrolled in time axis (**right**).

Long short-term memory is a type of recurrent neural network. The difference from the feedforward neural network mentioned earlier is that the RNN receives an input and generates an output while passing the output back to the input, so that each recurrent neuron can have two sets of weights: one is the current input, and the other is the output of the previous time step (frame), as shown in Figure 2. Because of this characteristic, it can be stated that the RNN has a certain memory ability. In the neural network, the composition that can retain a certain state beyond the time step (frame) is called a memory cell.

**Figure 3:**
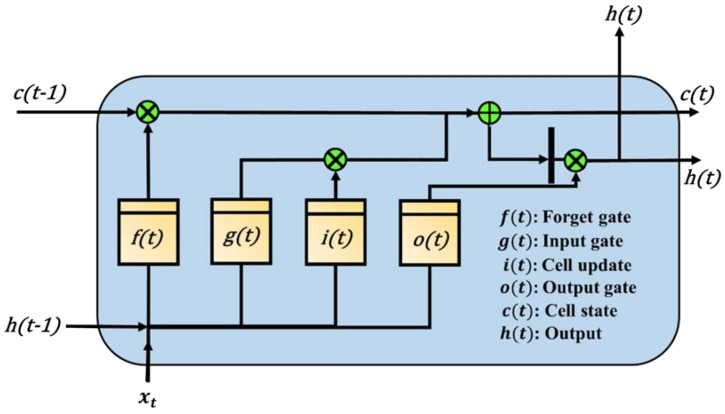
LSTM basic unit architecture.

However, the RNN also has a disadvantage. The former information has less influence on the latter. When more time series pass, the influence of the former information is almost zero. LSTM can improve the above-mentioned problems. LSTM consists of four units, which are the input gate (represented by g(t)), memory cell (represented by i(t)), output gate (represented by o(t)), and forget gate (denoted by f(t)). When the data are the input, the input valve controls whether to input the value this time and perform the operation or to control the memory cell to memorize the calculated value for use in the next operation. The state weight of this cell is c(t). The output valve controls whether to output the result of this operation, which is h(t). If not, the output is 0. Finally, the forgetting valve controls whether to clear the storage unit. The “control or not” of the four units are all learnable parameters in the network [38]. The actual LSTM operation unit is shown in Figure 3.

### 2.3. Reinforcement Learning

Reinforcement learning is a deep learning training method based on rewarding expected behavior and punishing bad behavior. In reinforcement learning, the goal is to develop an agent that improves its learning performance based on its interaction with the environment. In general, reinforcement learning agents are able to perceive and interpret the state of their environment, take action, and learn through trial and error. In reinforcement learning, a method of rewarding desired behaviors and punishing negative behaviors is designed. This feedback is not the correct true label or value, but a reward function that measures the quality of its calculation. This method assigns positive values to the desired behavior to encourage the agent and negative values to the bad behavior. This causes the main program to seek long-term and maximum block rewards for achieving the best solution. These long-term goals help prevent the subject from stagnating on smaller goals. Over time, subjects learn to avoid punishing factors and seek rewarding factors. However, in reinforcement learning, through interactions with the environment, agents can use reinforcement learning methods to learn a series of data calculus processes to maximize rewards through exploratory trial and error or deliberate planning [37]. We introduced three reinforcement learning algorithms below.

#### 2.3.1. Monte Carlo Learning

Monte Carlo reinforcement learning refers to the estimate of the true state directly from the complete-state sequence (episode) without knowing the state transition probability of the Markov decision process (MDP) value (expected value), and considers that the value of a state is equal to the average of all returns calculated from this state in multiple state sequences. A complete episode starts from a certain state. The individual interacts with the environment until the end state, and the environment gives the reward of the end state. The complete-state sequence does not require that the initial state must be a specific state, but requires that the individual finally enters a certain termination state recognized by the environment.

The Monte Carlo method is based on the method of averaging the samples of the return. In order to ensure that the previously defined return can be obtained, here, we specified the Monte Carlo method for episodic tasks. That is to say, we assumed that the experience was divided into multiple state sequences, and all state sequences eventually terminated. The estimate and policy for the value function would not change until the entire sequence of states is completed. Therefore, Monte Carlo method can be regarded as being episode-by-episode rather than step-by-step. The Monte Carlo feature is that it does not rely on state transition probabilities and learns directly from the complete-state sequence experienced. The central idea of its algorithm is to replace the expected value with the average return. Theoretically, the more complete-state sequences there are, the more accurate the prediction results.

#### 2.3.2. Q-Learning

Q-learning is a model-free, value-based off-policy learning algorithm. Model-free means an algorithm that estimates its optimal policy without any transformation or reward function from the environment. Q-learning updates its value function based on mathematical equations or a value-based approach, such as the Bellman equation, instead of estimating the value function using an ε-greedy policy. In the off-policy algorithm, the target prediction value updated by the neural network is calculated using actions that may be different from the actions actually observed in the future, and the weights $\bar{W}$ of the neural network need to be learned through training.

Training and prediction are performed at the same time, and the calculated values are used to update the weights and perform the next calculated action. It is tempting to choose the action with the largest Q value as the relevant prediction. However, this approach may underexplore the search space. Therefore, to choose the next action, the optimality prediction is combined with a strategy such as the ε-greedy algorithm. The action with the largest predicted payoff is chosen with probability (1 − ε). Otherwise, a random action is chosen. Therefore, the target predicted value of the neural network is calculated using the best possible action in the Bellman equation. This is why Q-learning is called an off-policy algorithm, in which the target predictions updated by the neural network are computed using actions that may differ from the actions actually observed in the future.

#### 2.3.3. SARSA (State–Action–Reward–State–Action)

SARSA (state–action–reward–state–action) is an updated algorithm of Q-Learning, in which the next optimal reward is not used to calculate the update. Conversely, the next step is to use the same ε-greedy policy update to obtain the target value for the operation at the next update of the weights. In Q-learning, the parameters are updated with the best possible action in each state, even though the actual executed policy may be greedy (encouraging exploration). In SARSA, the action actually selected by the ε-greedy method is used to perform the update. Therefore, this method is an on-policy method. Off-policy methods such as Q-learning can decouple exploration from exploitation, while on-policy methods cannot. Note that if we were to set the value in the ε-greedy policy to 0 (i.e., vanilla greedy), then both Q-learning and SARSA would focus on the same algorithm. However, because there is no exploration, this method does not work well. Therefore, SARSA is practical when learning cannot be conducted separately from the prediction.

## 3. Realizing In-Vehicle Fault Diagnosis

As far as the development of diagnostic technology in the past goes, the fault diagnosis of rotating machinery systems mainly relies on sensors to collect relevant physical analog signals, and, then, from a large amount of historical data, based on relevant theoretical values and actual manual experiences, the specific data thresholds that can be referenced for each part, i.e., the threshold value or the trend value of a specific change period, are set, so that the system can perform a predictive diagnostic analysis based on the characteristic calculation. If an intelligent sound diagnosis health degree system can be applied, and the acoustic characteristics of vehicle driving and human physiological representation are analyzed, the system continuously feedbacks the calculation to determine whether to issue a warning or not, and to compare the recognition results by voiceprint to evaluate the driving in real time. The status safety and system status diagnosis further realize the initial fault diagnosis and prediction of the system, and greatly improve the safety guarantee for vehicle driving.

Recently, the application of the IoV, which has attracted much attention, has become a key research topic. Research related to the fault identification and diagnosis of mechanical components has been published [26,39,40], and the main process usually relies on a large number of sensors installed on the work equipment. In the same way, a similar concept is applied to the diagnosis of driving status. Acoustic sensors are used to collect time series analog signals, and through feature extraction and the adaptive deep learning algorithm model, the health status of vehicles and driving can be analyzed, where the model data are cross-compared and output performance is able to present diagnostic information for the acoustic health status assessment. Supplemented with the above-mentioned application implementation framework, in the early stage when the potential risk of driving has not yet expanded, the location of components or driving conditions that may endanger safety can be predicted as soon as possible, and the driver should be immediately warned to turn their attention to the inspection unit for inspection and maintenance as soon as possible. The motivation comes from the goal to reduce the incidence of vehicle accidents as much as possible, and to continue to move towards the goal of zero accidents in traffic environments.

Various technology companies have also developed their own independent diagnostic devices for determining the characteristics of individual operating systems in different vehicles. There are millions of vehicles in the world. In view of this, in order to improve the quality of rapid maintenance, domestic and foreign repair shops have specially developed so-called professional automotive diagnostic instruments, such as “STAR Diagnosis” used by Mercedes-Benz (Shenzhen Automan Technology, Guangdong, China) [41], “CONSULT” by Nissan (NISSAN, Shenzhen Automan Technology, Guangdong, China) [42], “TECH 1” by Toyota (TOYOTA, Tech One Toyota Specialists, Kent, UK) [43], etc., all capable of independent diagnoses for their special models. The general structure of the system is to perform a signal transmission according to the sensors on the vehicle to obtain the basis for its fault judgment, and a bottleneck encountered at present is that its sensing signal only corresponds to an abnormality in a certain area. It cannot effectively improve its fault identification rate and realize an optimal fault diagnosis system.

Therefore, the diagnostic instruments currently used by major automakers can only provide a single piece of information, providing maintenance personnel with a judgment method to understand the appropriate diagnostic direction and accumulate their manual experiences, so as to deeply analyze the possible root cause of the failure. Another maintenance method is to use the accumulation of experience to listen to the position of abnormal noise when the machine is running. The above two maintenance and diagnosis methods are basically the diagnosis results generated by the accumulation of experience, analysis, and judgment supplemented by instrumental judgment. However, such inspection methods have a high probability of misjudgment and the process is cumbersome and time-consuming. Therefore, in order to improve the maintenance efficiency, diagnostic efficiency, and driving safety, the introduction of the intelligent vehicle audio diagnosis system is necessary. It can perform actual identification of the characteristics of mechanical faults or human health in a timely manner, so as to improve the safety of the overall driving environment as a whole.

### 3.1. Unsupervised Learning

The literature collection and discussion related to unsupervised methods are summarized as follows, as shown in Table 1.

#### 3.1.1. K-Means

The authors of [39] used R language as a platform and used the K-means algorithm to classify the characteristics of vibration signals by collecting normal and abnormal vibration signals from power fans of industrial furnaces. However, according to the conclusion from [39], the K-means failed to identify the new fan state and machine off-state, and the method had poor identification performance. The authors of [44] used Euclidean distance. The vibration signals were grouped into normal, warning signs of impending abnormality, and abnormality.

#### 3.1.2. PCA (Principal Component Analysis)

In [39], the PCA method was applied to reduce the dimensionality of the original data to facilitate the classification of the subsequent K-means method. The authors of [44] applied the PCA to classify and compare vibration fault signals. In [44], this statistic could be used to measure the value threshold, and any value above the threshold could be concluded as out-of-control data. Since the PCA is an algorithm that reduces the dimensionality of data while preserving most of the variation information in the dataset [45], in a simple context, it is an algorithm that identifies patterns in data to demonstrate their similarities and differences [46]. The authors of [47] proposed a fast self-compensation method for high-accuracy resistance simulation testing, and by using the PCA method, the response and accuracy were both improved in resistance testing.

#### 3.1.3. GAN (Generative Adversarial Network)

The authors of [48] applied the social generative adversarial network (GAN) to predict pedestrian trajectories. Social GANs have three main challenges, namely, the interpersonal and social acceptance and multimodel. Social GANs propose a recurring sequence-to-sequence model that aggregates information from different groups of people by observing motion history and predicting future behavior by applying a pooling mechanism. The authors of [49] utilized GANs to predict the future travel paths of vehicles on the road. The authors of [40] proposed a GAN to mitigate mutual interference in FMCW/CS (frequency-modulated continuous wave/chirp sequence) radars. The work was the first to use a GAN as an interference mitigation method for a RFFT spectrum in automotive radar systems. Experimental results showed that the proposed method had advantages in recovering the RFFT (real fast Fourier transform) spectrum compared with AR (autoregressive) and zeroing methods in terms of the SINR (signal-to-interference-plus-noise ratio).

The authors of [50] proposed simulating 3D LiDAR intensity for rendering using a GAN and described an efficient hash projection method to convert 3D point clouds into spherical projection images. Since iterative forecasting may lead to the accumulation of errors in multistep time series forecasting, in the study of [51], a direct forecasting strategy was adopted, which meant simultaneously forecasting multiple future steps in one forecast. In addition, because the detection range was becoming longer and longer, two methods were also proposed to improve the performance of sequence anomaly detection. First, inspired by the success of generative adversarial networks (GANs) in image generation [52,53] and sequence generation tasks [54,55], and, second, to adequately capture normal patterns in time series, the authors of [51] adopted the model built based on information from the time and frequency domains, and a new network architecture was designed in order to combine the discriminator with the sequence predictor. The discriminator tries to distinguish between real and predicted sequences. The authors of [51] also designed a new loss function that combined prediction loss with adversarial loss to train sequence predictors.

**Table 1:** Unsupervised learning.

Ref.	Method	Main Application
[44]	K-means PCA	A research study on unsupervised machine learning algorithms for early fault detection in predictive maintenance
[26]	K-means PCA	Fault class prediction in unsupervised learning using model-based clustering approach
[39]	GAN	Vehicle trajectory prediction based on social generative adversarial network for self-driving car applications
[40]	GAN	Automotive radar interference mitigation based on a generative adversarial network
[50]	GAN	Simulated intensity rendering of 3D LiDAR using generative adversarial network
[51]	GAN	Intelligent fault diagnosis under small sample size conditions via bidirectional InfoMax GAN with unsupervised representation learning
[56]	GAN	KfreqGAN: unsupervised detection of sequence anomaly with adversarial learning and frequency domain information
[47]	PCA	A quick self-compensation method of resistance simulation system in vehicle chassis dynamometer based on kernel PCA
[22]	GAN	A generative-adversarial-network-enabled deep distributional reinforcement learning for transmission scheduling in the Internet of Vehicles
[57]	GAN	SA-SGAN: a vehicle trajectory prediction model based on generative adversarial networks

The designed loss function enabled the end-to-end training of the above-mentioned two modules. Due to the limitations of data-driven intelligent fault diagnosis methods used for mechanical equipment under small sample conditions, the authors of [56] developed an unsupervised representation learning method for fault diagnosis under small sample conditions. Specifically, the samples were first fed into an encoder to obtain feature representations. Next, in order to alleviate the overfitting effect under the condition of small samples, based on implicit data augmentation in the GAN framework, the feature space of the encoder was calibrated and the mapping relationship was strengthened. The authors of [22] proposed a deep distributed Q-network (DDQN, also known as double-deep Q-learning) based on a SDN (software-defined network) using GAN to achieve efficient transmission scheduling in highly complex IoV environments. The paper proposed a GAN with a deep distributional RL for learning optimal policies for intelligent transmission scheduling in CIoV. In particular, the GAN’s approximation is applied to the action-value distribution to avoid the negative effects of noise and randomness compared to a classical DQN.

The authors of [57] proposed a new SA-SGAN (self-attention social generative adversarial network) prediction model, which utilized a self-attention mechanism to assign different weights to each feature of the trajectory sequence. It could effectively solve the problem of losing important information due to a long input sequence.

### 3.2. Supervised Learning

The literature survey related to supervision was summarized as follows, as shown in Table 2.

#### 3.2.1. SVM (Support Vector Machine)

In [58], to avoid the complexity of designing observers for model-based fault diagnosis methods, the authors investigated the problem of the fault diagnosis of steering actuators in autonomous vehicles with a model-based support vector machine (SVM) classification method. To this end, a two-degree-of-freedom (2-DOF) vehicle model was used to estimate the motion data of the autonomous vehicle, and the residual vectors between the measured and estimated signals were used as training data. The authors of [59] proposed a connected cruise control (CCC) framework considering the probability of merging the behavior of side vehicles. The control strategy of this framework first used vehicle-to-vehicle communication and radar recognition algorithms to detect surrounding vehicle objects. Next, the merging potential of sidecars was determined with the combination of a fuzzy support vector machine and sliding window detection.

After that, a cruising distance strategy considering the cut-in probability of merging behavior was proposed and applied to the CCC system to realize the braking of the main vehicle in the early stage. Since the SVM algorithm had some mixed and missing point problems in multiclass classification, the fuzzy SVM mixed fuzzy factors to improve the classification accuracy. The results showed that the method in [59] could accurately detect the lane-changing intention of a side car, so as to realize the advanced braking of the target vehicle when the side car changed lanes. The authors of [60] collected sound signals of vehicle tires in 12 different situations, and used SVM and MLP (multilayer perceptron) to classify and identify fault conditions. The main experimental procedure of [61] was divided into two parts. The first part focused on the extraction of vehicle power system fault signals, signal preprocessing, and background noise cancellation. The characteristic signal would be enhanced and extracted, and the temporal signal would be converted from a time domain to a frequency domain. The second part was mainly to establish a database of spectral characteristic signals, which could be used as training data methods for various machine learning methods.

The authors of [62] proposed a new ADAS (advanced driver assistance system) preprocessing algorithm to improve the accuracy of classifying drivers’ lane-change intentions by adding basic measurements from traditional in-vehicle sensors. The information about the vehicle state and road surface condition was augmented by using an artificial neural network (ANN) model, and the augmented information was fed back to a support vector machine (SVM) to detect the driver’s intention with high accuracy. The goal of [63] was to improve the classification accuracy of any given supervised learning algorithm by using the available unlabeled information in order to distinguish the proposed method from existing methods. The authors of [63] designed a meta-semisupervised learning algorithm that surrounded the underlying supervised algorithm and used unlabeled data to improve its performance. This problem is especially important when training supervised learning algorithms, while at the same time applying a limited amount of labeled information and large amounts of unlabeled data.

**Table 2:** Supervised learning.

Ref.	Method	Main Application
[55]	SVM	Fault diagnosis of an autonomous vehicle with an improved SVM algorithm subject to unbalanced datasets
[56]	SVM	Safety cruise control of connected vehicles using radar and vehicle-to-vehicle communication
[64]	LSTM	Vehicle driving behavior predicting and judging using LSTM and statistics methods
[61]	Decision tree (random forest)	Multiple-instance learning with random forest for event log analysis and predictive maintenance in ship electric propulsion system
[57]	SVM	Acoustic-based and machine-learning-driven methods for vehicle fault classification
[58]	DNN SVM	Implementation of machine learning for fault classification on vehicle power transmission system
[59]	SVM	Prediction of driver intention to lane change by augmenting sensor information using machine learning techniques
[62]	Decision tree (random forest)	Predicting the need for vehicle compressor repairs using maintenance records and logged vehicle data
[60]	SVM	SemiBoost: boosting for semisupervised learning
[63]	Decision tree (random forest)	Decision approach for maintenance of urban rail transit based on equipment supervision data mining
[65]	LSTM	Multivariate deep learning approach for electric vehicle speed forecasting
[66]	LSTM	Identification of driver braking intention based on long short-term memory (LSTM) network
[67]	Linear regression	Modeling vehicle-merging position selection behaviors based on a finite mixture of linear regression models
[68]	Linear regression	Measurement-based VLC channel characterization for I2V communications in a real urban scenario
[69]	Neural network	A multimodality fusion deep neural network and safety test strategy for intelligent vehicles
[70]	Neural network	Battery thermal runaway fault prognosis in electric vehicles based on abnormal heat generation and deep learning algorithms
[71]	Neural network	Object classification using CNN-based fusion of vision and LIDAR in autonomous vehicle environment

#### 3.2.2. Decision Tree

The authors of [64] provided an efficient solution to the problem of irregular, unbalanced, and unlabeled data, where conventional methods were outdated. A balanced random forest model was developed for unbiased classification and regression. The holistic approach recursively learned ungiven data labels while training the base learner. The authors of [65] proposed a data-driven method for predicting the impending failure of commercial vehicle air compressors. Predictive models were derived from currently available warranties and recorded vehicle data. These data sources are in-production data designed for and generally used for other purposes. This presented challenges that were presented, discussed, and addressed for building predictive models. The research contribution was twofold: the practical realization of these practical data, which is a rich type in the automotive industry, and the techniques to develop and test the processing, with inconsistent datasets, imbalanced and noisy class labels, and multiple per-vehicle feature selection, for example.

The author of [66] discussed the characteristics of comprehensive equipment maintenance and its defects in operation, and summarized the needs and development direction of intelligent decision-making in urban rail transit. Data mining and the analysis of monitoring systems can provide effective solutions for maintenance decision support. Aiming at the characteristics of mechanical equipment failures in urban rail transit (URT), the method proposed by the authors of [66] could identify the main equipment types for preventive maintenance by adopting clustering and decision tree rules. The preselected classes were evaluated from the perspective of discrete alarm data and continuous variables to demonstrate the validity of the results.

#### 3.2.3. LSTM (Long Short-Term Memory)

The authors of [67] proposed a new cognitive prediction method that used LSTM neural networks and a threat-level calculation method to assess whether nearby vehicles were dangerous. Using predictions from the first module and the evaluation from the second module, the vehicle’s AI system would determine the right decision. In [68], a data-driven approach to speed up prediction using univariate and multivariate techniques was emphasized, also proposing an LSTM-based model, which was trained on the dataset generated by the traffic simulator. The model was built on real-world urban trip data and calibrated using validated urban traffic profiles to evaluate the model’s efficiency in terms of short- and long-term speed prediction accuracy. In this study, two LSTM models were tested to predict vehicle speed. In [68], the authors presented the accuracy of the multivariate model and the univariate model. The experimental results showed that the multivariate model outperformed the univariate model due to the highly nonlinear factors of the velocity fluctuations, which were included in the dataset used for the multivariate model specification. The SVM-RFE (support vector machine–recursive feature elimination) algorithm used by the authors in [69] was used to select the feature parameters of the braking intent recognition model. The LSTM-based Gaussian hidden Markov model (GHMM) was used to identify light braking, normal braking, and hard braking under different time windows. The results showed that the recall rate and accuracy rate of the LSTM-based braking-intent recognition model were both above 95%.

#### 3.2.4. Linear Regression

In order to study the heterogeneity of drivers’ behavior in merging lane location selection, the authors of [70] proposed a finite mixture of linear regression models, which were constructed by combining drivers into lanes. The behavior was divided into three parts: gap selection, merging position selection, and merging execution or lane changing. The results presented in [70] demonstrated that the proposed model could successfully identify heterogeneity among merge drivers and yield accurate predictions of desired merge locations. In addition, this model could also be used to classify the driving styles of different drivers, which would be a breakthrough in alleviating traffic accidents and traffic jams caused by improper lane changes or merging.

The authors of [71] introduced an extensive measurement experiment of VLC (visible-light communication) channel characterization for I2V (infrastructure-to-vehicle) communication. Traffic lights were used to carry out the experiment. The three models proposed by the authors of [71] placed the visible-light receivers at three different heights—automotive headlights, dashboards, and interior mirrors. The authors of [71] compared the proposed model with the traditional Lambertian model in terms of accuracy and complexity to describe VLC channels in real urban situations. The results showed that the proposed architecture was more accurate than the Lambertian model in describing the VLC transmission from the traffic light to the vehicle compared to the architecture using the Lambertian model. The authors of [71] finally implemented specific hardware for modulating traffic light LEDs through the proposed model to provide drivers of different vehicle types with an easier view of traffic lights.

#### 3.2.5. Neural Network

Multimodal fusion based on deep neural networks (DNNs) is one of the important application methods for smart vehicles. The peculiarities of DNNs lead to problems with AI safety and security testing. In [72], the authors first proposed a multimodal fusion framework, called the integrated multimodality fusion deep neural network (IMF-DNN), which could flexibly accomplish these two objectives for the detection of the predicted steering angle, velocity, and end-to-end driving strategies. Then, the author proposed a DNN safety testing strategy, which systematically analyzed the robustness and generalization ability of the DNN under a large number of different driving environment conditions. It consisted of a neural network baseline for each modality and a central network for integrated multimodal fusion. Due to its architecture, central fusion network node, and loss function, the IMF-DNN had good flexibility and generalization ability. In [73], the authors proposed an enabled battery thermal runaway prediction scheme. It included temperature prediction using an improved CNN-LSTM neural network model and abnormal heat generation (AHG) diagnosis using a model-based scheme (MS). First, the memory cell of LSTM was modified and further combined with the CNN to extract temporal and spatial features of real-world factors related to battery temperature evolution. A vehicle-state driving behavior local weather analysis, PCA, and RAOM (random adjacent optimization method) were proposed to optimize the model input and hyperparameters of the improved CNN-LSTM model, thereby improving the modeling accuracy. The authors of [74] proposed an object classification method for the fusion of vision, light detection, and ranging (LIDAR) for autonomous vehicles in the environment. The method was based on the application of a convolutional neural network (CNN) and image upsampling theory. This approach was also adopted to guarantee object classification accuracy and minimal loss. The experimental results demonstrated the effectiveness and efficiency of the object classification strategy. In the training phase, using LIDAR information could accelerate feature learning and the convergence of the CNN on the target task prediction.

### 3.3. Reinforcement Learning

The literature survey related to reinforcement learning was summarized as follows, as shown in Table 3.

#### 3.3.1. Q-Learning

In [75], the authors described how to use the DQN method to manage the automatic lane change function of an autonomous vehicle, and used the Maxmin Q-learning method to study the vehicle’s self-driving system. This method improved the speed and stability of the vehicle in autonomous driving mode under certain conditions. However, the model used therein had significant drawbacks. In the case of complex and aggressive modes of other vehicle owners, vehicle safety still faces challenges. The authors of [76] introduced a reinforcement-learning-based decision-making method for intelligent autonomous vehicles for oncoming overtaking scenarios. The goal of reinforcement learning is to learn how to determine optimal decisions in the corresponding observations by interacting with the environment, using a reward function to estimate whether the decision was good or not. Dual-deep Q-learning (double DQN) agents were used to learn control policies for longitudinal speed and lane-change decisions. A prioritized experience replay (PER) was used to accelerate policy convergence. A two-way three-car situation with oncoming traffic was established in the simulation of urban mobility (SUMO) to train and test the strategy.

In [77]. the authors studied the real-time V2G (vehicle-to-grid) control problem under the conditions of hourly changes in electricity rates. The study’s model was inspired by the DQN algorithm, which combines the popular Q-learning with deep neural networks. A dual DQN model was proposed, maintaining two different networks to select or evaluate an action. The double DQN algorithm was used to control the charge/discharge operations in the hourly available electricity bill to maximize profits for EV owners throughout the parking time. Experimental results showed that the proposed method could effectively function in the real charging market, and it could significantly increase the business opportunity profit compared with other state-of-the-art EV charging schemes. In addition, the uncertainty of vehicles cutting from adjacent lanes makes it difficult for a vehicle’s automatic speed control strategy to determine judgments and effective control decisions. The authors of [78] proposed an autonomous driving system that established an intelligent speed control strategy for uncertain cut-in scenarios. The strategy was based on the Q-network to judge the cut-in action of the surrounding vehicles according to the Q value of the state–action pair, and output the adaptive control action in the current environment. In addition, according to the analysis of the cut-in scene, the Q-network was trained based on a new reinforcement learning method called the experience selection deep Q-learning network (ES-DQN). The proposed ES-DQN was an extension of the double-deep Q-learning network (DDQN) algorithm, which included two parts: empirical screening and policy learning. Based on the experience screened in the experience-screening part, the learning method proposed in the paper could train an intelligent speed control strategy. The experimental results showed that the performance of the intelligent speed control strategy trained by the ES-DQN was better than that of the DDQN method and the traditional ACC (adaptive cruise control) strategy in the uncertain cut-in scenario. At the same time, by adjusting the weight value in the reward function, the system could achieve different control objectives.

#### 3.3.2. Monte Carlo Learning

The authors of [79] proposed a reinforcement-learning-based approach that combined reinforcement learning agents with a Monte Carlo tree search algorithm to reduce unsafe behavior. The proposed security reinforcement learning framework mainly consisted of two modules: a risk state estimation module and a security policy search module. Once the future state was deemed dangerous, calculated by the dangerous state estimation module using the information of the current state and the output of the reinforcement learning agent, the MCTS-based security policy search module would be activated, and through the risk actions would add a bonus (reward) to ensure a safer exploration. The authors of [79] used a secure reinforcement learning algorithm to solve the decision-making problem in a two-lane overtaking scenario. It combined traditional reinforcement learning algorithms with a search-based Monte Carlo tree search algorithm to add additional negative rewards to actions that may involve dangerous driving behaviors, thereby speeding up the algorithm’s convergence. The authors of [80] introduced a general framework for tactical decision-making that combined planning concepts with learning in the form of Monte Carlo tree search and deep reinforcement learning. The method was based on the AlphaGo Zero algorithm and extended it to domains with continuous state spaces and where self-play could not be used. The framework was applied to two different highway driving cases in a simulated environment, and it showed better performance than commonly used baseline methods.

The topic of [80] was tactical decision making, which considered high-level decisions that adapted the behavior of the vehicle to the current traffic situation [81,82]. For example, these decisions could handle when to change lanes, or whether to stop at an intersection. The authors of [80] proposed a general framework based on the AlphaGo Zero algorithm, which combined the following concepts: planning and learning to create a tactical decision-making body for autonomous driving. Planning was conducted through a variant of Monte Carlo tree search, which built a search tree based on random sampling. The difference between a standard MCTS (Monte Carlo tree search) and the version used here was that the neural network biased the sampling towards the most relevant parts of the search tree. Neural networks were trained through reinforcement learning algorithms, where the MCTS component not only reduced the number of training samples required, but also helped to find long-term temporal correlations. The proposed framework was applicable to two conceptually different driving cases and outperformed commonly used baseline methods.

The results in [80] showed that the proposed framework, combining planning with learning, could be used to create tactical decision-making agents for autonomous driving. For two conceptually different highway driving cases, the generated agents performed better than either planning (in the form of MCTS) or learning (in the form of a trained neural network) alone. The agent also outperformed baseline methods based on the IDM (intelligent driver model) and MOBIL (minimizing overall braking induced by lane) models. The proposed framework was flexible and could be easily adapted to other driving environments. It was also sample-efficient and required an order of magnitude fewer training samples than a DQN subject applied to similar cases [83]. The author of [84] took the steady state of motion as the starting point, first establishing a simplified model of the supercavity vehicle, completing the theoretical analysis according to the actual state, and obtaining the relationship expression between the angles of the supercavity vehicle when it was in stable motion.

**Table 3:** Reinforcement learning.

Ref.	Method	Main Application
[75]	DQN	Lane change of vehicles based on DQN
[76]	DQN	Decision-making for oncoming traffic-overtaking scenario using double DQN
[77]	DQN	Integration of electric vehicles in smart grid using deep reinforcement learning
[78]	DQN	ES-DQN: a learning method for vehicle intelligent speed control strategy under uncertain cut-in scenario
[79]	Monte Carlo learning	Safe reinforcement learning for autonomous vehicle using Monte Carlo tree search
[80]	Monte Carlo learning	Combining planning and deep reinforcement learning in tactical decision making for autonomous driving
[84]	Monte Carlo learning	Simulation of supercavitating vehicle steady motion
[9]	SARSA	Autonomous RL: autonomous vehicle obstacle avoidance in a dynamic environment using MLP-SARSA reinforcement learning
[85]	SARSA	Power management strategy of hybrid vehicles using SARSA method
[86]	SARSA	A path-planning algorithm based on RRT and SARSA (Ȝ) in unknown and complex conditions

Secondly, according to the theoretical analysis, the simulation of the smooth motion of the supercavity vehicle was programmed and calculated, and the simulation result of the smooth motion of the supercavity vehicle was tabulated, from which the force of the supercavity vehicle was deduced. The simulation results were processed, and the simulation results were compared with the test results. In the study of the stable motion of the supercavity vehicle, it was feasible to use this method to analyze the structure of the supercavity vehicle.

#### 3.3.3. SARSA (State–Action–Reward–State–Action)

MLP-SARSA is a policy-based reinforcement learning method that draws information and rewards from the environment to help autonomous vehicles avoid dynamically moving obstacles. Compared with other traditional reinforcement algorithms, MLP with SARSA provides significant advantages over dynamic environments. Therefore, the authors of [9] proposed a multilayer perceptron SARSA (MLP-SARSA)-based reinforcement learning method for dynamic obstacle detection and avoidance for automatic vehicle navigation. The experimental results of [9] showed that the trained MLP-SARSA could drive self-driving cars with more confidence in dynamic environments when comparing two reinforcement learning algorithms—Q-learning and MLP-SARSA. The authors of [85] proposed a power management strategy based on the SARSA algorithm. In the architecture of [83], the controller of a gasoline–electric hybrid vehicle was designed as a learning agent in a reinforcement learning method.

Using the urban dynamometer driving schedule (UDDS) database as a framework and applying an environmental interaction and trial and error, the optimal power management strategy between fuel power and battery power was found to not only maintain the state of charge of the battery, but also achieve the optimum fuel consumption. With the SARSA algorithm, the agent learned at each time step which action would lead to the highest reward and maximized its reward in the long run. Experimental results showed that the SARSA algorithm could achieve better performance than Q-learning. To solve the local path planning problem of the wheeled mobile robot in unknown and complex conditions, the author of [86] proposed a rapidly exploring random tree (RRT) path optimized based on the reinforcement learning SARSA algorithm. The planning algorithm is known as the RL-RRT (reinforcement learning–rapidly exploring random tree) method in [86]. The RL-RRT method utilizes the task return function, the objective distance function, and the angle limit to ensure the randomness of the RRT, reduce invalid nodes, and optimize the performance of the RRT algorithm. This architecture reduces the sampling blindness of the RRT algorithm to a certain extent, improves the avoidance efficiency, and seeks to solve the local path-planning method.

## 4. Overview of the Application of Vehicle Fault Diagnosis to the Internet of Vehicles (Using Acoustic Signals to Achieve Fault Prediction as an Example)

### 4.1. Overview of Vehicle Transmission Acoustic Signal Processing

The system parts of the vehicle are composed of many rotating structures. Under the wear and tear of long-term driving and operation, the relevant components experience functional abnormalities due to temperature, pressure, metal fatigue, wear, and tear [5]. If the failure factor of the rotating element is not detected early, it can be a great threat to driving safety. As far as the field of acoustics research is concerned, the application of voiceprint recognition in automobile fault prediction is a very novel technology, but there are still many technical obstacles to overcome [60], such as complex sound field feature filtering, voiceprint data collection, digital filtering algorithms, and the selection of machine learning training models. Acoustic sensors are used to measure abnormal vehicle signals and thereby determine the abnormal characteristics of vehicle parts. Usually, when parts are in the early stage of failure, the signal-to-noise ratio of the characteristic signals obtained is very low. Therefore, it is difficult to detect and deal with. As a result, high-resolution sensors must be used for measurement. Generally speaking, when a vibration signal is sensed for a general system component, it is almost damaged in the middle, late stage, or even soon after the system component fails and stops running. At this time, the vehicle is also damaged, which may be severely affected and fail to break down. The most easily damaged part in the vehicle is the bearing [87]. If there is a problem with the bearing and it is not detected in time, it may cause the damaged steel ball or the entire bearing to wear, which increases the clearance between the shaft and the bearing, causing a serious imbalance in the rotating system. Even traffic accidents occur due to damage to the universal joint bearings of important systems.

In the early stage of bearing damage, abnormal signals are formed at sound wave frequencies ranging from 20 kHz to 100 kHz. As the damage of the bearing increases, the frequency of the abnormal signal gradually appears in the low-frequency domain. The effect is not ideal [88]. For example, to collect vehicle fault acoustic signals, microelectromechanical array microphones of smart phones can be used [89]. Compared with vibration signals, microphone signals are more suitable for sensing fault signal characteristics in the early stage of rotating machinery failure, due to features such as a wide-frequency response, high sensitivity, and good stability. For any physically measurable signal, the signal must be preprocessed to obtain a better characteristic acoustic signal. According to the research in [7] by M. M. Hasan et al., the background ambient broadband noise is the majority among the measured acoustic signals. Background noise includes human noise or other noises in the environment during measurement. If the unprocessed acoustic signal is extracted for features, the filtered result is not likely to be ideal. For example, the preadding processing is performed to strengthen the signal part of the specific high-frequency band of the speech. The original signal collected by the vehicle must contain the noise formed by the friction and collision of many different parts. It is also necessary to filter and eliminate the unwanted noise in order to obtain a stable characteristic target signal for acoustic feature data annotation.

**Figure 4:**
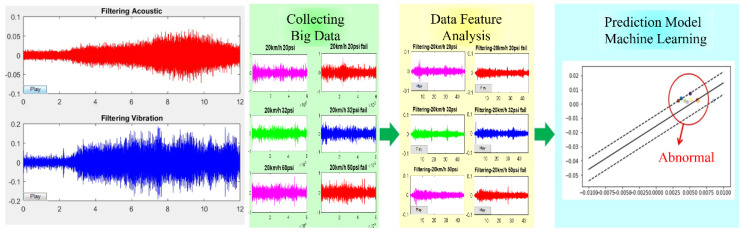
Schematic diagram of vehicle dynamic voiceprint recognition.

**Figure 5:**
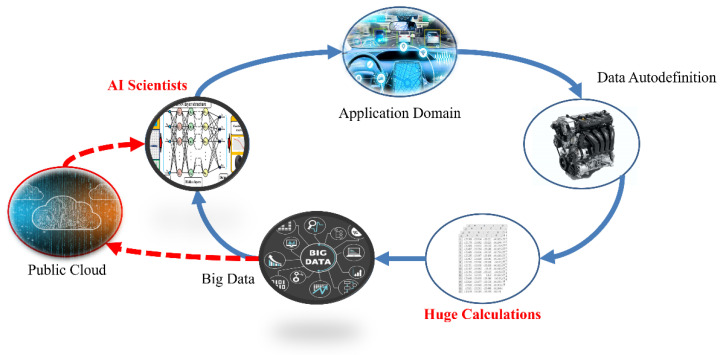
Application of IoV in AI architecture diagram.

In addition to the key technology of acoustic sensing big data collection, how to construct a predictive model with a high-recognition rate and short training time is an important research topic for vehicle diagnosis systems. The characteristic data model must also match the most suitable learning algorithm parameters. In the process of acoustic signal filtering, the spectrum analysis is a fault feature diagnosis method commonly used for acoustic feature identification. After collecting the acoustic signals of various vehicle fault conditions, the acoustic signals can be imported into various digital filtering algorithms such as LPC [90], MFCC [60,61], and wavelet transform (WT). The time-domain signal is converted into a spectrum and the background noise is removed, where the key features are preserved. This is called “feature extraction”. Then, the data are marked to facilitate the subsequent import of the dataset into various machine learning algorithms for classification and identification. Figure 4 shows the flow chart of the identification of a vehicle dynamic voiceprint feature. Figure 5 shows the architecture diagram of the AI IoV.

**Figure 6:**
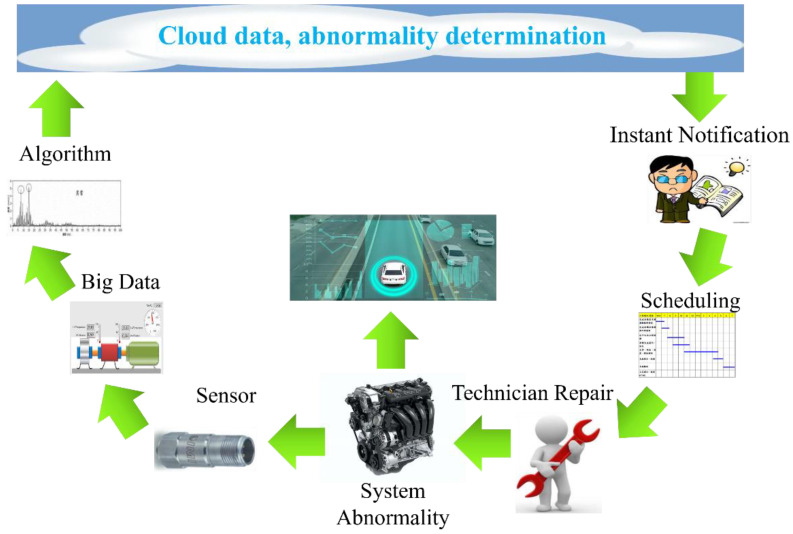
Schematic diagram of IoV fault prediction combined with cloud computing architecture.

In any situation, the vehicle may fail without warning. According to the current diagnostic technology, it can only be predicted through regular in-plant maintenance inspections or on-board diagnostic systems, and the exact cause of the failure needs to be determined by the automaker’s technicians. The above-mentioned process of vehicle diagnosis status belongs to the category of reactive maintenance and preventive maintenance. Accidents often happen unexpectedly. To achieve real-time fault prediction and fault-type diagnosis, the sound signals collected by the sensor are digitally filtered and marked with data through cloud computing, and then identified using machine learning algorithms, followed by comparing existing fault conditions. A cross-comparison can immediately remind the driver to enter the factory for inspection when the signs of failure are detected, and even send a warning to the neighboring vehicles in combination with the IoV system, which can greatly reduce traffic accidents caused by mechanical failures. The architectural concept is shown in Figure 6.

### 4.2. Establishment of Fault Diagnosis Model

The modeling of the fault prediction architecture is divided into three parts, where the first part is the feature extraction of the acoustic signal of the automobile fault. The acoustic sensor is used to collect various signals, such as the cabin, engine, chassis, and tires, including various signals of normal driving and possible abnormal fault states. The original data are used to filter and extract the audio signal of various fault signals. The second part is to mark a large number of acoustic datasets of various styles, establish a voiceprint feature spectrum dataset when the vehicle is driving dynamically, complete the original sound file signals of various states, and then filter the sound signals for each state through various digital filtering algorithms. These steps used to obtain spectral features are called feature extractions. After all the features are marked with data, the voiceprint feature dataset of the recognition model is established. Finally, a machine learning model is applied to train and validate the feature dataset.

**Figure 7:**
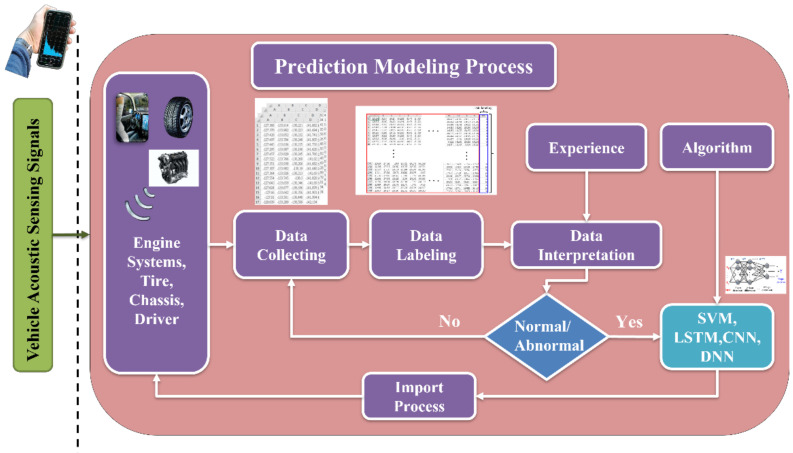
Malfunction acoustic diagnosis prediction modeling process.

The third part applies various deep learning architectures for complete deep neural network training, verification, and identification. The dataset uses various machine learning and deep learning methods for model training and performance comparison, and classifies and identifies the spectral characteristics of voiceprints in various states. After data interpretation, normal and abnormal sounds can be identified and classified. In addition to using various commonly used deep learning methods, such as DNN and CNN, if the scale of the dataset is multipattern and the signal is a continuous time series, the long short-term memory model (LSTM) can be used to solve the long-term sequence high-dimensional, gradient disappearance, and gradient explosion during the training process. The modeling process of vehicle acoustic fault diagnosis is shown in Figure 7.

### 4.3. Multiple Data Source Input Architecture

**Figure 8:**
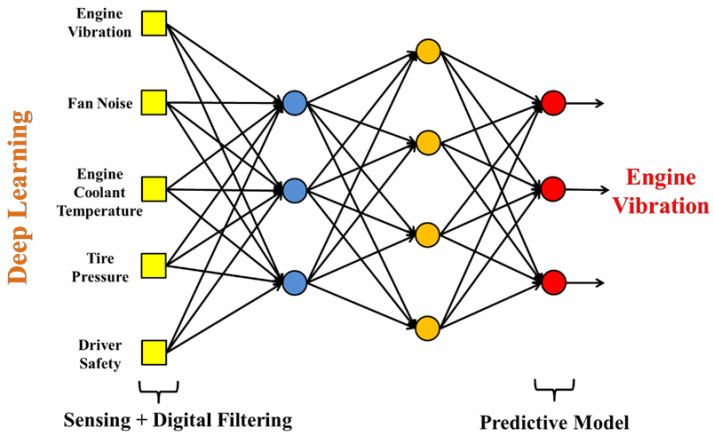
Multiple data source input architecture.

Due to the wide variety of signals that can be sensed on a vehicle, the multi-input model architecture proposed here can be used. In the block diagram of Figure 8, after various types of input data are collected by sensors and digital filtering architecture, these data can be distributed and parallelized with various machine or deep learning algorithms, followed by obtaining the results. In addition to the above-mentioned physical data that can provide sensing, this architecture can also be extended to other sensing data, such as vehicle radar images, the frequency and times of stepping on the brake or accelerator, battery charge, and discharge conditions, etc. Therefore, the structure of Figure 8 is to input various types of signals into the weight calculation of each hidden layer to the node, and compare the various types of prediction outputs. The difference after comparison is fed back to the system to improve its closed-loop identification effect, thereby giving the system optimal control.

## 5. Discussion and Conclusions

In the current era of 5G communication development, the IoV communication network is a very important development focus. In recent years especially, the rapid development of unmanned vehicle technology has also strengthened the necessity of vehicle fault diagnosis. Immediate and adequate maintenance operations are critical to the operation of autonomous vehicles, as they can provide warnings and emergency proactive protective actions before failures occur, minimizing surprises caused by mechanical failures. To be able to warn of failures before they occur, predictive maintenance (PdM) can be used, which is a preventative optimal maintenance strategy that predicts potential failures and takes immediate and appropriate maintenance actions [14]. Due to the more cost-saving advantages of predictive maintenance, it is gradually replacing traditional periodic maintenance strategies, including reactive maintenance and preventive maintenance. In recent years, with the rapid development of sensor and network technology, the availability of condition monitoring data for various electromechanical equipment, such as acoustics, vibration, temperature, and pressure, has increased significantly. With the rapid development of big data and parallel computing processors, AI technology, especially the wide application of machine learning and deep learning, has been optimally applied in current predictive maintenance systems. In addition, the AI technology used in driverless vehicles can also be used for lane identification, electric vehicle charging optimization, vehicle trajectory prediction, vehicle transmission scheduling, and driving behavior prediction. It can be seen that AI technology is crucial to the development of the IoV.

Recently, numerous research articles on predictive maintenance have been published in the literature, including theoretical studies, implementation methods, and applications, such as industrial forecasting. This paper aimed to provide a brief overview of the recent literature review of AI on predictive maintenance techniques, especially in the field of automotive applications. Several studies have confirmed that the identification accuracy of using deep learning methods to predict faults is higher than using traditional machine learning techniques. However, compared with traditional machine learning techniques, deep learning methods require more raw data. The case studies analyzed demonstrated how machine learning can effectively predict failures or anomalies in a wide range of applications, and how it can improve the toolset for predictive maintenance [15]. Mixed models and physical models were selected for the most appropriate application, according to those demonstrated in the literature. In some cases, such as the process analyzed in [91], where a large amount of data was not available, the collection of available data was an extremely important process. Finally, the role of digital transformation technologies in predictive maintenance was analyzed. The digital transformation of vehicles enables automakers to better diagnose abnormal conditions and predict the remaining useful life of dismantled components, thereby improving vehicle performance and safety. In addition, next to failure prediction, artificial intelligence is often used in vehicle trajectory prediction and driver behavior prediction. These applications are not limited to a single vehicle, but combine vehicle-to-vehicle, vehicle-to-infrastructure, vehicle-to-passenger and even driver-to-vehicle applications.

Looking at those applications described above, we found that one of the main limitations of these contributions, also recognized in other reviews [15], was the collection of datasets for sensing real-world conditions. These datasets are often considered to be trade secrets of high importance by automotive companies. Due to the difficulty of obtaining the dataset, this does not allow the combination and comparison of new methods with existing techniques. Another limitation is that it is difficult to evaluate the effectiveness of developed methods by using real data. Real-world data are usually only partially labeled, whereas annotated data are time-consuming and require a lot of expertise to validate. However, in order to construct methods that produce more robust results, it is necessary to test models with labeled data, even if only to train them on unlabeled data. In addition, due to the unpredictability of actual vehicle failures and the complexity of road conditions, the production of datasets will is a huge project, and how to quantify the data of various sensing signals is also a big challenge.

There are two important points to consider when choosing an algorithm for a vehicle failure prediction model in terms of development and optimization. First, what is the best algorithm for a given problem? Second, what problems can the chosen algorithm solve? This paper analyzed state-of-the-art AI technologies demonstrated in the literature, keeping in mind their computing power, advantages, disadvantages, suitability for parallel processing, other algorithms, and processing time. According to our search and sorting, we found that all deep learning methods actually have their own uses. For the unsupervised learning method proposed, for example, K-means is suitable for industrial applications, and the PCA can help to reduce the dimension of the dataset, so that the dimension of the dataset can be reduced to facilitate the subsequent introduction of other machine learning algorithms. GANs can be used in mechanical failure prediction and lane analysis.

As part of the supervised learning method, linear regression is suitable for light signal identification and classification. Decision trees can handle problems of balance and data labeling. The SVM can be used not only to predict vehicle paths, but also to predict vehicle behaviors. Neural networks are the most widely used among these methods. As long as the data are quantized and the parameters are adjusted to create a dataset, it can be applied to many aspects. Finally, LSTM is suitable for vehicle speed sets and vehicle braking intention prediction.

There are three parts of reinforcement learning. The first is Q-Learning, which can manage self-driving lane changes, control vehicle speed, and optimize electric vehicle charging. Monte Carlo Learning can predict the behavior of other vehicles and reduce unsafe driving behaviors. It is also capable of predicting the force analysis of aircraft. SARSA can predict obstacle detection and avoidance, and can also be used for electric vehicle power management and robot control.

In recent years, numerous research articles on predictive maintenance, including theoretical studies and industrial applications, have been published in many scientific journals and research reports. These works aim to provide a brief overview of recent research contributions to predictive maintenance techniques, especially in the military aerospace field. From this literature, we learned how deep learning methods guarantee better fault prediction accuracy; on the other hand, deep learning methods require more data for computation than traditional machine learning techniques. The case studies analyzed in this article demonstrated how machine learning can effectively predict failures or anomalies in a wide range of applications, and how it can improve vehicle predictive maintenance models.

In the future, AI application research could solve the application of general predictive maintenance results in automobile cases. Under the possible application of deep learning reinforcement learning algorithm, the existing model could be trained to obtain another applicable actual data model with the same functionality, namely, the so-called “meta-learning”. “Meta-learning” is used to acquire the learning ability for new jobs from past learning. It is suitable for small samples and multitasking learning scenarios, and can solve the problem of rapid learning when new jobs lack training samples.

In terms of machine learning, it has not yet reached the standards of human learning at many levels, so we hope that we can use the skills of meta-learning to train machines to learn and judge from the original database through machine learning, so that when a new database is used, it can be verified from the past database. Additionally, testing different algorithm models should be on the same real dataset to compare the methods in the literature, followed by applying the best method to a vehicle or IoV system to further verify the effectiveness of the method and develop the most appropriate as well as safest active protection system or car-networking control system. Another future research direction is to develop and combine different AI model methods to obtain new application models in order to optimize existing technologies and provide more effective vehicle control system failure prediction analyses.

## Abbreviations

The following abbreviations are used in this manuscript:

2-DOF	Two-degree-of-freedom
ACC	Adaptive cruise control
ADAS	Advanced driver assistance system
AHG	Abnormal heat generation
AI	Artificial intelligence
ANN	Artificial neural network
AR	Autoregressive
CART	Classification and regression tree algorithm
CCC	Connected cruise control
CNN	Convolutional neural network
DNN	Deep neural network
DDQN	Double-deep Q-learning
DQN	Deep Q-learning network
ES-DQN	Experience selection deep Q-learning network
FMCW/CS	Frequency-modulated continuous wave/chirp sequence
FNN	Feedforward neural network
GAN	Generative adversarial network
GPS	Global positioning system
IDM	Intelligent driver model
IMF-DNN	Integrated multimodality fusion deep neural network
I2V	Infrastructure-to-vehicle
IoT	Internet of Things
IoV	Internet of Vehicles
LPC	Linear predictive coding
LSTM	Long short-term memory
MCTS	Monte Carlo tree search
MDP	Markov decision process
MEMS	Microelectromechanical system
MFCC	Mel-frequency cepstral coefficient
ML	Machine learning
MLP	Multilayer perceptron
MOBIL	Minimizing overall braking induced by lane
MS	Model-based scheme
MSE	Mean square error
PCA	Principal components analysis
PdM	Predictive maintenance
PER	Prioritized experience replay
RAOM	Random adjacent optimization method
RBF	Radial basis function
RFFT	Real fast Fourier transform
RL	Reinforcement learning
RL-RRT	Reinforcement learning rapidly exploring random tree
RNN	Recurrent neural network
RRT	Rapidly exploring random tree
SARSA	State–action–reward–state–action
SA-SGAN	Self-attention social generative adversarial network
SDN	Software-defined network
SSE	Sum of square error
SVM	Support vector machine
SVM-RFE	Support vector machine–recursive feature elimination
SUMO	Simulation of urban mobility
UDDS	Urban dynamometer driving schedule
URT	Urban rail transit
V2G	Vehicle-to-grid
VFDD	Vehicle fault detection and diagnosis
VLC	Visible light communication
WT	Wavelet transform
